# Stacked Sparse Auto-Encoders (SSAE) Based Electronic Nose for Chinese Liquors Classification

**DOI:** 10.3390/s17122855

**Published:** 2017-12-08

**Authors:** Wei Zhao, Qing-Hao Meng, Ming Zeng, Pei-Feng Qi

**Affiliations:** School of Electrical and Information Engineering, Tianjin University, Tianjin 300072, China; 2015203156@tju.edu.cn (W.Z.); qh_meng@tju.edu.cn (Q.-H.M.)

**Keywords:** stacked sparse auto-encoders, electronic nose, deep learning, Chinese liquors classification

## Abstract

This paper presents a stacked sparse auto-encoder (SSAE) based deep learning method for an electronic nose (e-nose) system to classify different brands of Chinese liquors. It is well known that preprocessing; feature extraction (generation and reduction) are necessary steps in traditional data-processing methods for e-noses. However, these steps are complicated and empirical because there is no uniform rule for choosing appropriate methods from many different options. The main advantage of SSAE is that it can automatically learn features from the original sensor data without the steps of preprocessing and feature extraction; which can greatly simplify data processing procedures for e-noses. To identify different brands of Chinese liquors; an SSAE based multi-layer back propagation neural network (BPNN) is constructed. Seven kinds of strong-flavor Chinese liquors were selected for a self-designed e-nose to test the performance of the proposed method. Experimental results show that the proposed method outperforms the traditional methods.

## 1. Introduction

In recent years, with the improvement of people’s living standard, people pay more and more attention to food safety. Chinese liquors industry is a unique traditional light industry in China. However, counterfeit and fake liquor problems have been plaguing the industry and consumers. How to identify Chinese liquors quickly and accurately is an urgent problem to be solved. Traditional testing instruments, such as chromatograph and spectrometer, are expensive, bulky and inconvenient to carry, and difficult to realize rapid detection [1].

Electronic nose (e-nose) is a portable and rapid instrument inspired by olfactory systems of mammals, which has been widely used in food safety, environmental monitoring and disease diagnosis [2,3,4]. Data processing procedure of a traditional e-nose mainly consists of several steps: pre-processing, feature extraction (generation and reduction) and classification. Each step has plenty of optional methods or algorithms, and different choices may lead to different identification results. For example, Jing et al. [5] presented a new combination method for Chinese liquor classification. In the step of feature generation, ten features were selected based on information theory. In the procedure of feature reduction, they have tested performance of two methods: kernel entropy component analysis (KECA) and kernel principle component analysis (KPCA). Finally, they presented a multi-linear classifier and used the back propagation neural network (BPNN) as well as the linear discrimination analysis (LDA) for comparison. More recently, Jia et al. [6] proposed a new hybrid algorithm for Chinese liquors classification, in which man-made features were reduced using a combined KECA-LDA technique and the extreme learning machine (ELM) was applied as a classifier. Before they found this optimal KECA-LDA-ELM combination algorithm, they have tried some other combination algorithms, such as KECA-BPNN, KECA-ELM, and KECA-LDA-BPNN. Obviously, in the traditional design of an e-nose, obtaining optimal combination method is complex and time consuming.

The motivation of our work is to simplify the traditional data-processing steps for e-noses using deep learning (DL) techniques. The concept of deep learning (DL) was first presented by Hinton and Bengio in 2006 [7,8], to solve the optimization problems of deep network structure. The stacked sparse auto-encoder (SSAE) was a kind of DL model, which was presented by Hinton and Ranzato et al. [9,10]. In the past few years, DL has been successfully applied to image recognition [11], speech recognition [12], and target recognition [13]. DL techniques break the limitation of layer numbers and overcome the gradient dilution and the local minimum problems in traditional neural networks [14,15]. To our knowledge, DL methods have not been widely used for data processing of e-noses. Längkvist and Loutfi [16] applied deep belief networks (DBN) and conditional restricted Boltzmann machine (CRBM) for an e-nose to identify the special bacteria in blood and agar. Längkvist et al. [17] used two unsupervised feature learning methods: stacked restricted Boltzmann machines and stacked auto-encoders, for fast classification of meat spoilage markers. More recently, Liu et al. [18] implemented the DL technique to tackle the sensor drift problem and then to improve the classification performance of the machine olfaction system.

As far as we know, there is no report on DL based e-noses for Chinese liquors recognition. In this paper, we present a stacked sparse auto-encoder (SSAE) [19] based DL approach for Chinese liquors classification. We use the SSAE to learn inherent features automatically from the response curves of gas sensors in unsupervised manner. After that, the learned features are used for training a BPNN to classify different brands of liquors. The proposed method does not need the steps of preprocessing and feature extraction. In the experimental procedure, seven brands of strong-flavor Chinese liquors were selected to test the effectiveness of our proposed method. The results of the SSAE based method were compared with those of stacked auto-encoders (SAE) [20] based BPNN [21] and SSAE based support vector machine (SVM) [22] as well as two kinds of traditional methods.

The remainder of this paper is organized as follows: Section 2 presents the method description, including spare auto-encoder, SSAE based BPNN method and traditional methods applied to the data processing of the e-nose we designed. Section 3 describes the experiments and results. Finally, conclusions are given in Section 4.

## 2. Method Description

### 2.1. Sparse Auto-Encoder

**Figure 1:**
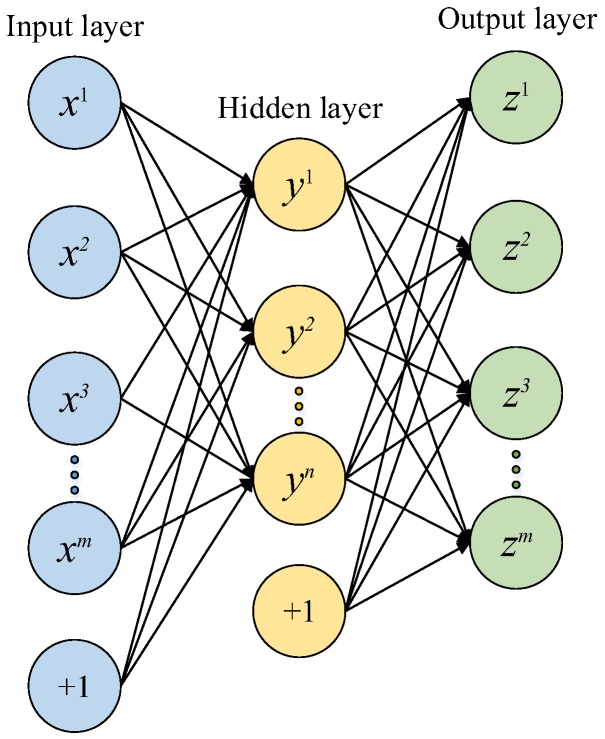
Structure of an auto-encoder neural network (“+1” represents the bias vector).

As an unsupervised learning algorithm, an auto-encoder network consists of three layers: input layer, hidden layer and output layer [23]. It makes the output layer equal to the input layer, which minimizes the reconstruction error to extract a best expression of the hidden layer. The auto-encoder network based data processing for e-noses consists of two steps (as shown in Figure 1): Firstly, the original e-nose data $x={\left[x^{\left(1\right)}x^{\left(2\right)}\cdots x^{\left(m\right)}\right]}^{T}$ is encoded from the input layer to the hidden layer (encoding):

(1) y=f(x)=S(Wx+b),

where $y={\left[y^{\left(1\right)}y^{\left(2\right)}\cdots y^{\left(n\right)}\right]}^{T}$ denotes the feature expression of the hidden layer; *m* and *n* are the total number of the nodes in the input and hidden layers, respectively. *W* is the weight matrix and *b* represents the bias vector. S(×) is the sigmoid function, which is expressed as $S(\times )=1/(1+e^{-\times })$. Secondly, the feature expression *y* is decoded from the hidden layer to the output layer (decoding):

(2) $$z=g(y)=S(W^{T}y+b^{\prime }).$$

The feature expression *y* is decoded to obtain the reconstructed vector $z={\left[z^{\left(1\right)}z^{\left(2\right)}\cdots z^{\left(m\right)}\right]}^{T}$, where *b*′ stands for the bias vector.

The sparse auto-encoder [24] is an extension of the auto-encoder, which introduces sparse restrictions to the hidden nodes, in order to control the number of activated neurons. The system complexity and parameters can be reduced due to less number of activated neurons, so that the sparse auto-encoder can learn better features [25]. The cost function of the sparse auto-encoder under the entire data set is expressed as:

(3) $$J=\frac{1}{m}\sum_{i=1}^{m}\left[\frac{1}{2}{\left(x^{\left(i\right)}-z^{\left(i\right)}\right)}^{2}\right]+\frac{\lambda }{2}\sum_{i=1}^{m}\sum_{j=1}^{n}{\left(w_{ij}\right)}^{2}+\beta \sum_{j=1}^{n}\left[\rho \log\frac{\rho }{\rho_{j}}+(1-\rho )\log\frac{1-\rho }{1-\rho_{j}}\right].$$

The first term is the square root error indicating the difference between the input and the output. The second term is the weight decay term used to solve the over-fitting problem, where λ is the weight attenuation coefficient and $w_{ij}$ is the weight corresponding to the input node *i* and the hidden node *j*. The last term is the sparse penalty term, where ρ stands for the sparse target value, β is the weight of the sparse penalty item, and $\rho_{j}$ is the average activation quantity of the hidden unit *j*. The back-propagation algorithm is adopted to obtain the parameters *W* and *b* by minimizing the cost function *J* [21], where the stochastic gradient descent approach is used for training. The parameters *W* and *b* in each iteration process can be updated as:

(4) $$w_{ij}=w_{ij}-\varepsilon \frac{\partial }{\partial w_{ij}}J,$$

(5) $$b=b-\varepsilon \frac{\partial }{\partial b}J.$$

where ε is the learning rate. A forward propagation algorithm is used to compute the average activation quantity $\rho_{j}$ to get the error, and then the back-propagation algorithm is applied to update the parameters *W* and *b*.

### 2.2. SSAE Based BPNN (SSAE-BPNN)

**Figure 2:**
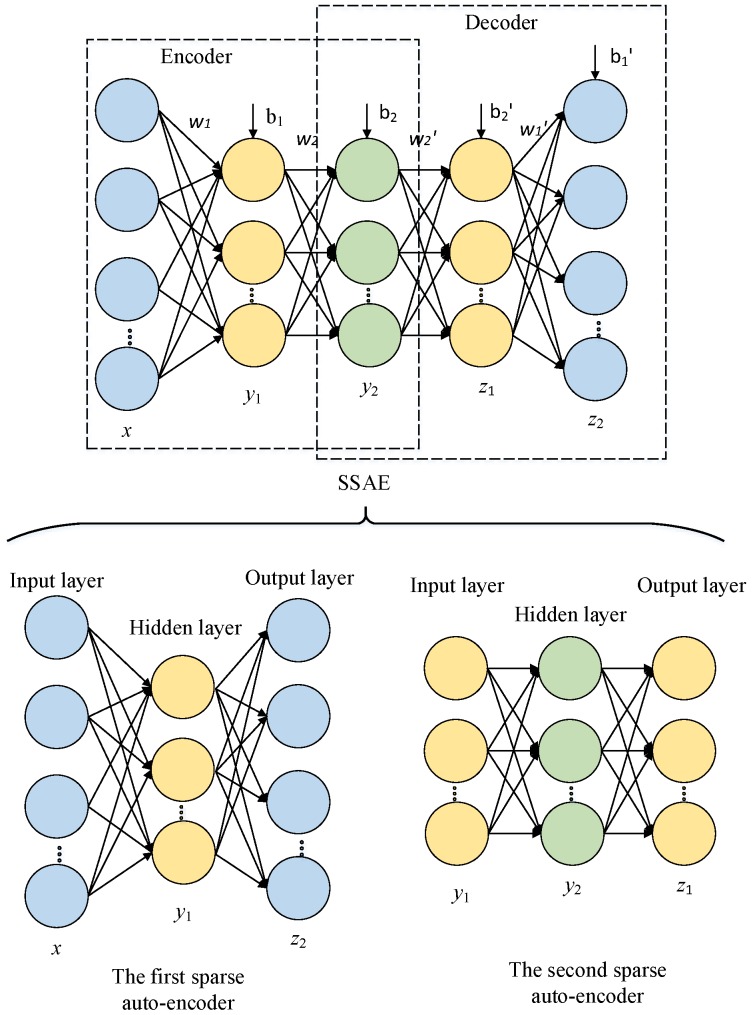
A stacked sparse auto encoder (SSAE) network composed of two sparse auto-encoders.

**Figure 3:**
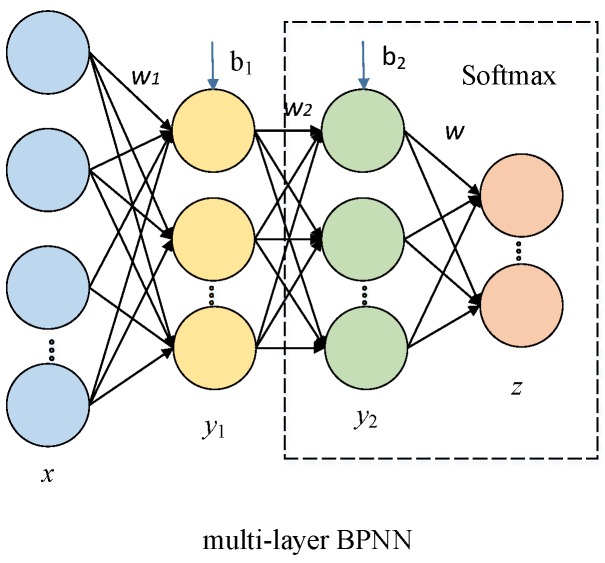
SSAE based multi-layer back propagation neural network (BPNN).

An SSAE is usually built with multiple sparse auto-encoders. Figure 2 shows an SSAE composed by two sparse auto-encoders, where the hidden layer of the first sparse auto-encoder is treated as the input layer of the second sparse auto-encoder. A greedy layer-wise unsupervised algorithm [18] is used to train each sparse auto-encoder independently. After the SSAE is trained to learn the features, the multi-layer BPNN is used for classification. Instead of direct utilization of learned features of SSAE, the parameters of SSAE are used to initialize the BPNN to get the features for classification. The steps of training the SSAE based BPNN are described as follows:

(1) Pre-training: The two sparse auto-encoders are trained in succession, and the SSAE parameters (*W* and *b*, cf. Equations (1) and (2)) are obtained after the pre-training step;
(2) Constructing BPNN: The encoder layers of SSAE is taken as the first three layers of the multi-layer BPNN, and the third layer (the second hidden layer of BPNN) is connected with the output layer which corresponds to the types of Chinese liquors in our study through Softmax regression (see Figure 3);
(3) Initialization: The network parameters of the first three layers are initialized with the pre-training parameters;
(4) Fine-tuning: The forward propagation algorithm is used to train the BPNN network, and then the parameters are fine-tuned using the back-propagation algorithm with the labels.

### 2.3. Data Processing Procedures Based on SSAE-BPNN and Traditional Methods

Two traditional methods are used to compare with the SSAE-BPNN method. The response curves of ten gas sensors are sampled at a frequency of 100 Hz.

The first traditional method is presented by Jing et al. [5]. The data processing procedure is described as follows: Firstly, the original response curves are preprocessed by wavelet denoising filters and conductivity normalization. Secondly, ten features for each response curve are chosen, they are (1) the time reaching the response maximum value; (2) root mean square; (3) arithmetic mean; (4) geometric mean; (5) harmonic mean; (6) the maximum value of the first-order derivative; (7) the time when the maximum value of the first-order derivative is reached; (8) the average differential; (9) the integration of the response curve when it reaches the maximum value and (10) the mean curvature. After feature generation, the dimension of the original feature space for ten response curves is 100, and then the dimension is reduced to 20 using KECA. Finally, the classification of Chinese liquors is implemented using BPNN and SVM.

**Figure 4:**
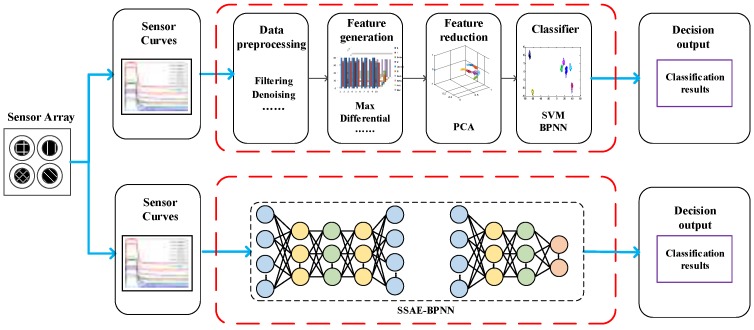
SSAE-BPNN vs. Traditional methods.

The procedure of the second traditional method can be illustrated by the upper part of Figure 4. Firstly, the original response curves of ten gas sensor are preprocessed using the Savitzky-Golay wave filter [26]. Secondly, we select five features for each response curve, including (1) the maximum value, (2) the maximum value of the first-order derivative, (3) the response value corresponding to the maximum of first-order derivative, (4) the minimum value of the first-order derivative and (5) the maximum value of the second-order derivative. The dimension of the original feature space for ten response curves is 50. Then, the number of features is reduced to 19 using the PCA method [27]. The extracted features are applied to classify the Chinese liquors using BPNN and SVM.

The features selection for traditional methods is usually manual, which is time-consuming and hard to be appropriate. Due to this reason, we presented a deep learning method to learn the features automatically instead of manual choice. Compared with the relatively complicated procedures of traditional method (see the upper part of Figure 4), the structure of the proposed SSAE-BPNN method (see the lower part of Figure 4) is quite concise. Firstly, we directly use the SSAE method to learn the features from the down-sampled response curves. Then the SSAE based BPNN method is applied to classify the Chinese liquors.

## 3. Experiments and Results

### 3.1. Experimental Materials

Seven kinds of strong-flavor Chinese liquors were used in the experiments, including Bianfenghu (BFH), Bainianwanjiu (BNWJ), Hongjinjiu (HJJ), Lanjinjiu (LJJ), Luzhoulaojiao (LZLJ), Mianzhudaqu (MZDQ), Niulanshan (NLS). Table 1 provides the detailed information for the seven kinds of Chinese liquors.

### 3.2. Electronic Nose

**Figure 5:**
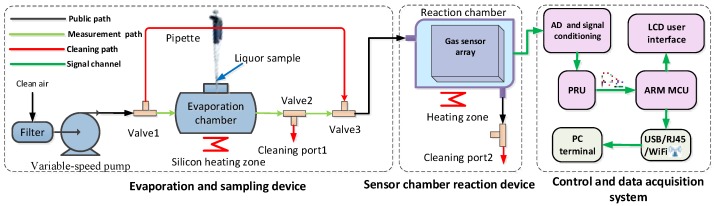
Structure of the designed e-nose system for Chinese liquors recognition.

**Figure 6:**
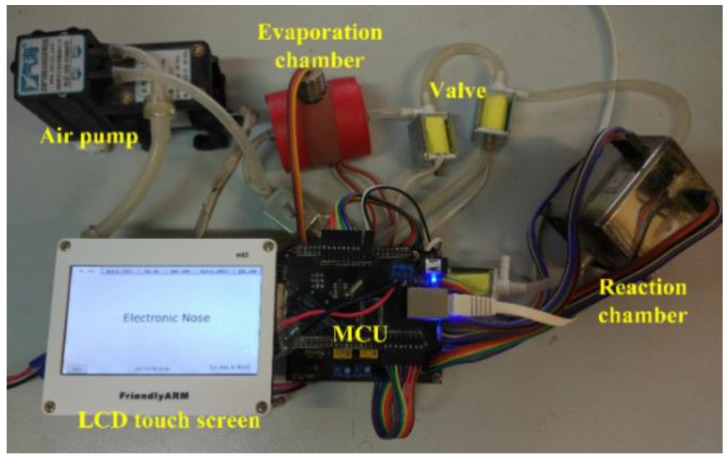
E-nose experiment platform.

The self-designed e-nose system [28] for Chinese liquors identification consists of three parts: liquor dynamic evaporation and sampling device, sensor chamber reaction device, control and data acquisition system (shown in Figure 5). A physical picture of the e-nose experimental platform is shown in Figure 6. Through the hardware and software design, the e-nose system can realize automated sampling scheme with a friendly user interface. After setting up the experimental condition parameters and dropping the liquor sample, the sampling process is implemented automatically. The sensor array consists of ten types of metal oxide semiconductor (MOS) sensors, which are TGS series (TGS2602, TGS2611, TGS2620, TGS880) from Figaro Engineering Inc. (Osaka, Japan), MiCS series (MiCS-5121, MiCS-5521, MiCS-5524, MiCS-5526) from SGX Sensortech Inc. (High Wycombe, Britain), and MP502 as well as WSP2110 from Winsensor Ltd. (Zhenzhou, China). MOS sensors have a shortcoming of slow recovery, especially for the online monitoring applications. To solve this problem, a reservoir computing approach could be used to overcome the slow temporal dynamics of MOS sensor arrays, allowing identification and quantification of chemicals of interest continuously and reducing measurement delays [29].

The experimental procedure is like this: 5 μL for each kind of Chinese liquors was dripped into the inlet of the evaporator chamber using a pipette gun. Then the evaporator chamber was heated to a constant temperature of 65 °C. After the liquid liquors were evaporated into gas, the sample was inhaled to the reaction chamber with an air pump, and the gas was contacted with the sensor array. Finally, the response curves were saved. After each experiment, the evaporator chamber and the reaction chamber were cleaned for 3 min and 1 min, respectively. The experimental process was repeated 30 times for each kind of Chinese liquors and totally 210 groups of data were obtained.

### 3.3. Parameters Setting for SSAE-BPNN

DeepLearn Toolbox [30] is an open source software library that includes popular machine learning and artificial intelligence techniques, such as Artificial Neural Networks (ANN), Convolutional Neural Networks (CNN), Stacked Auto-encoders (SAE) and Deep Belief Networks (DBN), etc. We utilized ANN and SAE algorithms in DeepLearn Toolbox to realize the SSAE-BPNN and added SVM algorithm to SAE in DeepLearn Toolbox to realize the SSAE-SVM.

**Figure 7:**
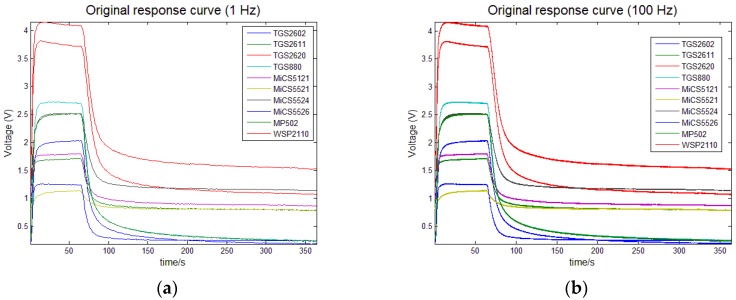
Response curves of the sensor array, (**a**) represents the original response curve (1 Hz); (**b**) represents the original response curve (100 Hz).

Figure 7 presents the recorded response curves of ten gas sensors with a sampling frequency of 100 Hz. The recorded time for each gas sensor was 364 s and the initial dimension of 100 Hz data (364,000) was too large as the input of the proposed method. So, the analyzed data were collected with an interval of 1 s, i.e., the recorded response data were down-sampled from 100 Hz to 1 Hz for the SSAE method to learn from. As shown in Figure 7, when the sampling rate was down-sampled to 1 Hz, the overall trend of response curves was not changed, which almost made no effect on the feature learning. Therefore, the total number of the analyzed data for each experiment was 3640 and then we chose 3640 nodes in the input layer of the constructed SSAE-BPNN.

**Figure 8:**
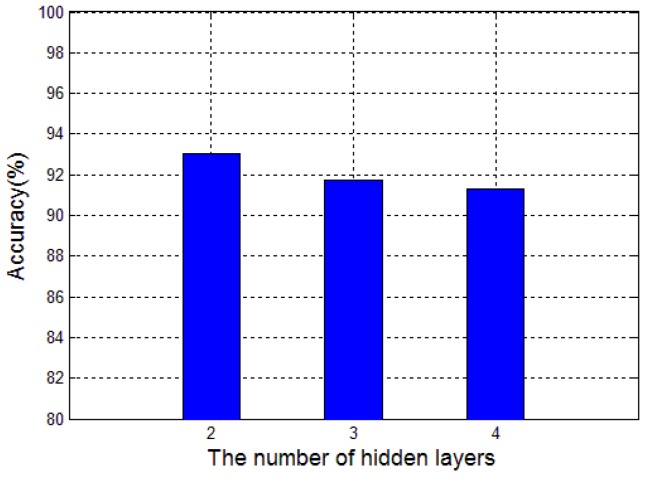
Influence of the number of hidden layers on classification accuracy.

To alleviate the possible over-fitting problems caused by small samples, we want to use a simple network structure to implement deep learning [30]. To achieve better classification results, we have carried out tests with different number of hidden layers and hidden-layer nodes for the SSAE. During the tests, the number of nodes in each hidden layer was set to 200, and the iteration times for both the pre-training and fine-tuning procedures were set to 10. Other parameters' settings were listed in Table 2. The test results showed that the classification rate cannot be effectively increased with an increase in the number of hidden layers. As shown in Figure 8, when the hidden-layer numbers are set 3 and 4, the classification accuracy are 91.71% and 91.28%, respectively, which are lower than 93.04% obtained with two hidden layers. Therefore, the number of the hidden layers for the SSAE was set to 2 in our subsequent study.

**Figure 9:**
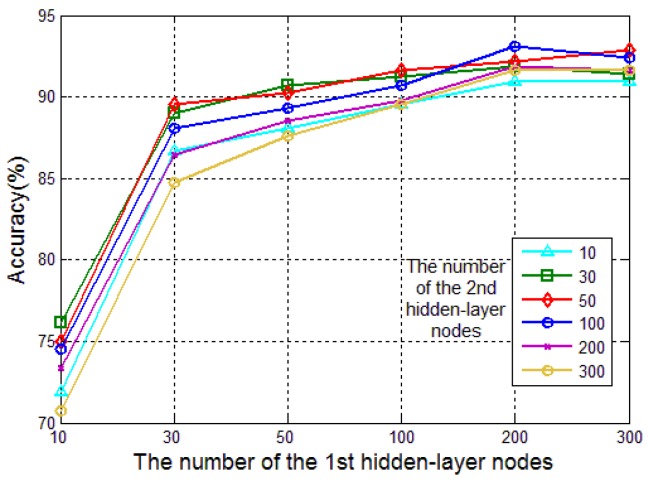
Influence of the number of hidden-layer nodes on classification accuracy.

Figure 9 illustrates the influence of the number of hidden-layer nodes on classification accuracy, where the horizontal ordinate is the number of the 1st hidden-layer nodes and the legend represents the number of the 2nd hidden-layer. When the numbers of two hidden-layer nodes were changed, the classification accuracy of different choice was given. The optimal settings for the number of nodes in the 1st and the 2nd hidden-layers were 200 and 100, respectively. Considering that there are seven kinds of Chinese liquors tested in the experiment, the number of nodes in output layer was set as 7.

**Figure 10:**
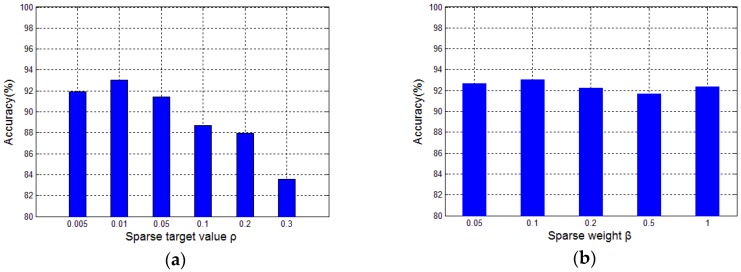
Influence of the SSAE parameters on classification accuracy, (**a**) shows the influence of the sparse target value on classification accuracy; (**b**) shows the influence of the sparse weight on classification accuracy.

Figure 10 shows the influences of the sparse target value ρ and the sparse weight β on the classification accuracy, from which the optimal settings for the both values were set to 0.01 and 0.1, respectively. Using the similar choice method, we set the learning rates for SSAE ($\varepsilon_{1}$) and BPNN ($\varepsilon_{2}$) to 0.1 and 1, respectively. All the parameter settings for the SSAE-BPNN are listed in Table 2.

### 3.4. Experimental Results and Discussion

#### 3.4.1. SSAE-BPNN

Considering that the samples number is small, we adopted the way of cross-validation to evaluate the performance of the proposed SSAE-BPNN and the traditional methods, where the cross-validation could also eliminate the over-fitting problems [31]. The 210 groups of data were randomly divided into ten groups, i.e., a ten-fold cross validation was used. When any one group was used as the testing set, the other nine groups were used as the training sets. The average of ten classification accuracy was taken as the final result. In this work, we adopted the 10-fold cross-validation rather than the leave-one-out cross-validation because the latter cost more time and had the same performance with the 10-fold validation.

**Figure 11:**
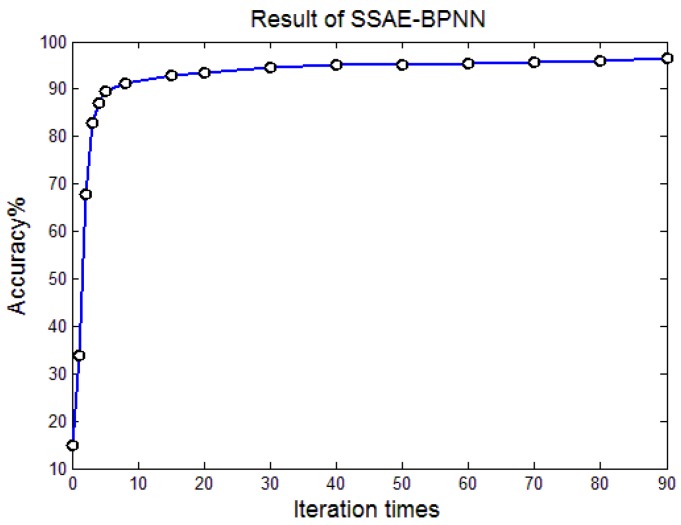
Influence of iteration times on classification accuracy.

The training process was divided into two steps. The first step is an unsupervised pre-training process, and the second step is a supervised fine-tuning process. Figure 11 presents the relationship between the iteration times and the classification accuracy, from which we can see that when the iteration times is 10, the classification accuracy exceeds 90%. With an increase in the iteration times, the accuracy increases slowly. When the iteration times reaches 90, the highest classification accuracy of 96.67% can be obtained.

**Figure 12:**
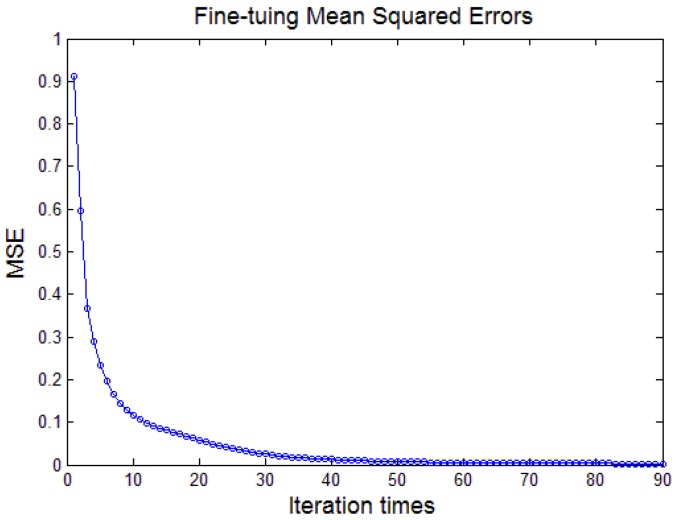
Relationship between the fine-tuning mean squared error (MSE) and iteration times.

Figure 12 shows the relationship between the mean squared errors (MSE) and the iteration times during the fine-tuning process. When the iteration times reaches 90, MSE converges to 0.

#### 3.4.2. SSAE-BPNN VS SAE-BPNN and SSAE-SVM

**Figure 13:**
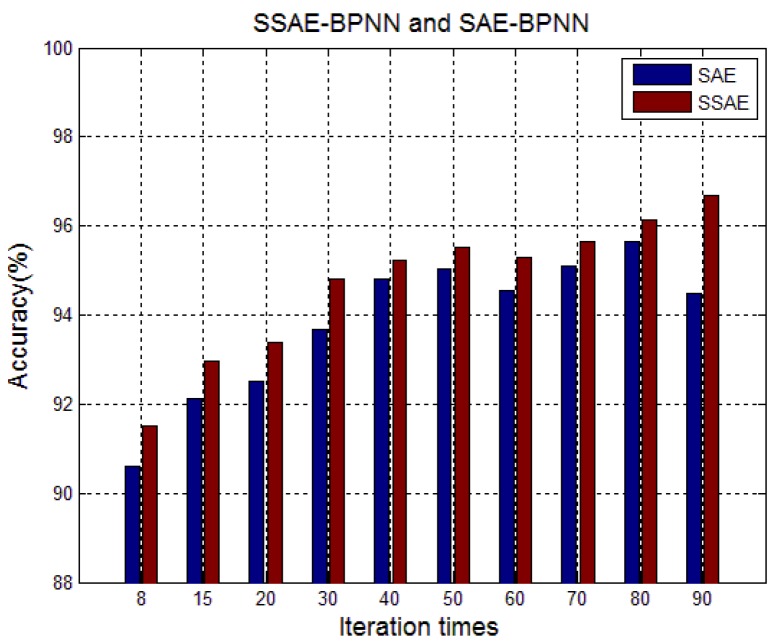
Comparison of SSAE-BPNN and SAE-BPNN.

In order to demonstrate the function of sparsity in the feature learning process, the performance of SAE and SSAE has been compared. As shown in Figure 13 and Table 3, at the same iteration times, the classification accuracy of SAE-BPNN is lower than that of SSAE-BPNN. In addition, the performance of BPNN and SVM was also compared.

**Figure 14:**
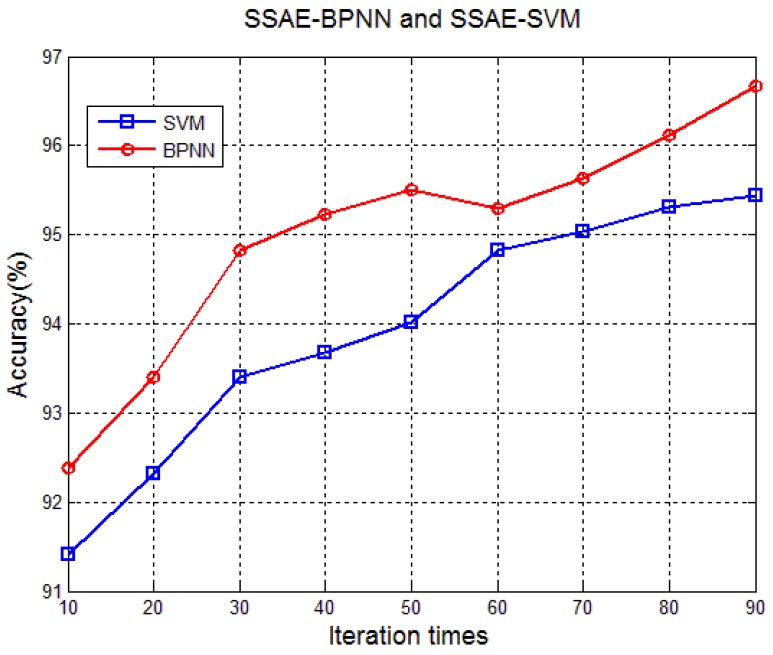
Comparison of SSAE-BPNN and SSAE-SVM.

An SSAE-SVM was also constructed for comparison. The SSAE-SVM used the features learned by the SSAE (the second hidden-layer output of the SSAE) as the input of the SVM classifier. For a fair comparison, here the SSAE was kept the same as the one used in the SSAE-BPNN, but the features learned by the SSAE was not fine-tuned during training for the SVM. The test results show that SSAE-BPNN is superior to SSAE-SVM, see Figure 14 and Table 4. From Table 3 and Table 4, we can find that the cross-validation interval of confidence for SSAE-BPNN is lower than both the SAE-BPNN and SSAE-SVM, which indicates that the SSAE-BPNN has a better classification precision.

#### 3.4.3. SSAE-BPNN VS Traditional Methods

**Figure 15:**
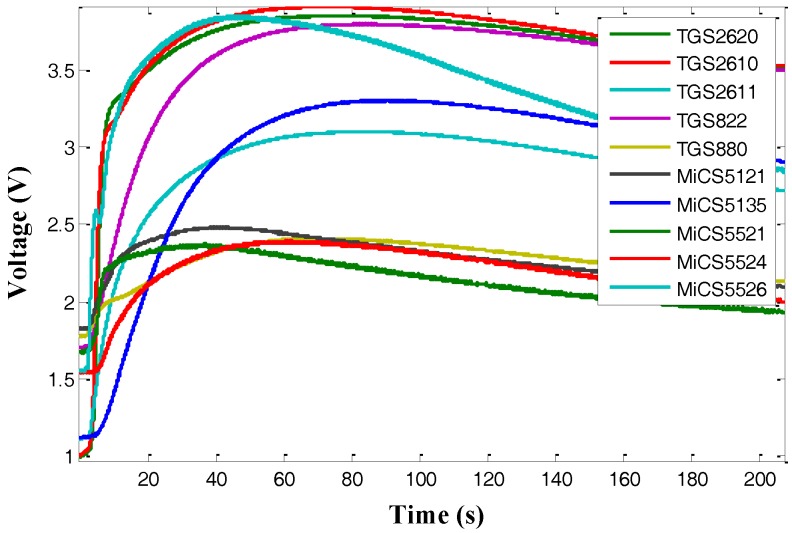
Response curves of the first generation e-nose platform.

**Figure 16:**
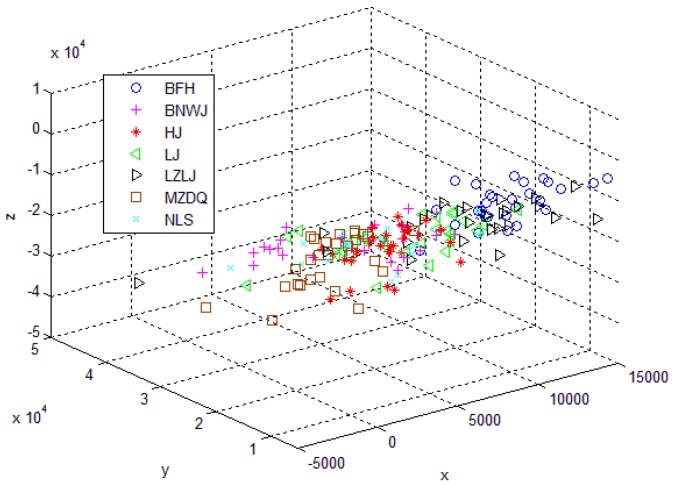
Three-dimensional principal component analysis (PCA) score plot.

The classification results are shown in Table 3. The first traditional method (the KECA based, cf. [5]) has been applied to process the first-generation e-nose data (see Figure 15), and obtained good classification results. However, when it was utilized to the second-generation e-nose data (see Figure 7), the results were not satisfactory. Thus, the second traditional method (the PCA based) was used for comparison. To get information about the data distribution, the projected three-dimensional PCA score plot using the second traditional method is shown in Figure 16. The recognition results based on the two traditional methods and the SSAE based method are presented in Table 5. The second PCA based traditional method is superior to the KECA-based method, but it is still inferior to our proposed SSAE-BPNN method. The employed time of each method is also listed in Table 5. It could be seen that the traditional methods cost more time due to the man-made feature extraction process. Although the SSAE-BPNN costs more time than the SSAE-SVM, it is worth to obtain better results.

## 4. Conclusions

A novel deep learning (DL) based data-processing method has been proposed for electronic noses (e-noses) to classify different brands of Chinese liquors. The proposed method utilizes a stacked sparse auto-encoder (SSAE) to directly learn the features from the gas sensors’ response data, and the learned result is then used to construct a new BPNN, i.e., SSAE-BPNN. By combining the SSAE with a support vector machine (SVM), an SSAE-SVM is also constructed. The SSAE based methods do not need the tedious and complicated steps used in traditional methods for e-noses, such as preprocessing and feature extraction (generation and reduction).

To verify and compare the classification performance of the proposed method as well as the traditional methods, seven kinds of strong-flavor Chinese liquors have been used as experimental materials using the self-designed e-nose. The results show that the SSAE-BPNN method achieves the highest 96.67% classification accuracy, which is superior to the results of SSAE-SVM and two kinds of traditional methods.

It is important to emphasize that traditional methods can obtain good classification results by optimizing the data-processing procedure, e.g., combinatorial feature extraction methods and classifiers. However, due to many choices in combination, the optimization process is normally empirical and complicated. The proposed SSAE based DL method can not only simplify the data processing process, but also can obtain good classification results.

In the future work, to realize rapid and on-line liquors recognition based on e-noses systems, we will try other deep neural network approach, like deep reservoir computing algorithm, to solve the problems of the slow temporal dynamics of sensor arrays.

## Figures and Tables

**Table 1:** Sample information of experimental liquors.

Name	Alcohol (Vol)	Raw Materials	Origin Place (China)
BFH	40% Vol	Sorghum, wheat, barley, pea	Tianjin
BNWJ	38% Vol	Sorghum, wheat, barley, pea	Anhui
MZDQ	38% Vol	Sorghum, rice, wheat, corn	Sichuan
LJJ	48% Vol	Sorghum, wheat, barley, pea	Tianjin
HJJ	38% Vol	Sorghum, wheat, barley, pea	Tianjin
NLS	42% Vol	Sorghum, wheat, rice	Beijing
LZLJ	38% Vol	Sorghum, rice, wheat	Sichuan

**Table 2:** Parameters setting up of SSAE-BPNN.

SSAE	Value	Definition	BPNN	Value	Definition
In	3640	No. of input-layer nodes	In	3640	No. of input-layer nodes
$S_{1}$	200	No. of 1st hidden-layer nodes	$S_{1}$	200	No. of 1st hidden-layer nodes
$S_{2}$	100	No. of 2nd hidden-layer nodes	$S_{2}$	100	No. of 2nd hidden-layer nodes
ρ	0.01	Sparse target value	Out	7	No. of output-layer nodes
β	0.1	Sparse weight	$\varepsilon_{2}$	1	Learning rate
$\varepsilon_{1}$	0.1	Learning rate			

**Table 3:** Accuracy and interval of confidence of SSAE-BPNN & SAE-BPNN.

Iteration Times	SSAE-BPNN		SAE-BPNN	
	Accuracy	Interval of Confidence	Accuracy	Interval of Confidence
8	0.915079	0.007976	0.906122	0.008116
15	0.926531	0.007706	0.921088	0.007706
20	0.934014	0.007493	0.92517	0.010546
30	0.948299	0.005786	0.936735	0.006572
40	0.952381	0.008248	0.948299	0.008878
50	0.955102	0.004647	0.95034	0.005399
60	0.953061	0.003286	0.945578	0.007199
70	0.956463	0.006406	0.95102	0.011571
80	0.961224	0.005786	0.956463	0.006406
90	0.966667	0.003888	0.944898	0.008632

**Table 4:** Accuracy and interval of confidence of SSAE-BPNN & SSAE-SVM.

Iteration Times	SSAE-BPNN		SSAE-SVM	
	Accuracy	Interval of Confidence	Accuracy	Interval of Confidence
10	0.923810	0.006148	0.914286	0.009524
20	0.934014	0.007493	0.923129	0.01853
30	0.948299	0.005786	0.934014	0.010075
40	0.952381	0.008248	0.936735	0.011571
50	0.955102	0.004647	0.940136	0.014511
60	0.953061	0.003286	0.948299	0.011143
70	0.956463	0.006406	0.95034	0.009858
80	0.961224	0.005786	0.953061	0.018934
90	0.966667	0.003888	0.954422	0.011939

**Table 5:** Recognition results and employed time based on traditional methods and DL methods.

Algorithm	Traditional Method 1		Traditional Method 2		DL Methods	
	KECA-BPNN	KECA-SVM	PCA-BPNN	PCA-SVM	SSAE-BPNN	SSAE-SVM
Accuracy (%)	70.47	72.86	90.47	91.90	96.67	95.44
Time (s)	892	872	587	567	59	39
